# Transarterial radioembolization versus chemoembolization for hepatocellular carcinoma: a meta-analysis

**DOI:** 10.3389/fonc.2024.1511210

**Published:** 2025-01-17

**Authors:** Wenxiao Lu, Tongsheng Zhang, Fengfei Xia, Xiangzhong Huang, Fulei Gao

**Affiliations:** ^1^ Department of Gastroenterology, Jiangyin Hospital affiliated to Nantong University, Jiangyin, China; ^2^ Department of Interventional Radiology, Jiangsu Hospital of Huocheng County, Huocheng, China; ^3^ Department of Interventional Medicine, Binzhou People’s Hospital, Binzhou, China; ^4^ Department of Interventional Radiology, Jiangyin Hospital affiliated to Nantong University, Jiangyin, China

**Keywords:** hepatocellular carcinoma, transarterial chemoembolization, transarterial radioembolization, yttrium-90, meta-analysis

## Abstract

**Background:**

Currently, inoperable hepatocellular carcinoma (HCC) is treated by both transarterial radioembolization (TARE) and transarterial chemoembolization (TACE). However, their relative efficacy and outcomes remain unclear. This meta-analysis aimed to compare TARE and TACE to evaluate their safety and efficacy in treating inoperable HCC patients.

**Methods:**

Relevant studies were identified by searching the Web of Science, PubMed, and Wanfang databases. Pooled analyses were used to compare treatment response rates, complications, and overall survival (OS) outcomes between the TARE and TACE groups.

**Results:**

This analysis selected 8 studies comprising 1026 and 358 patients that respectively underwent TACE and TARE treatment. The results revealed that the TARE group had significantly higher pooled total response, disease control, and 1-year OS rates compared to the TACE group (P = 0.04, 0.003, and 0.02, respectively), with a corresponding increase in OS (P = 0.0002). Furthermore, rates of complications including fever and abdominal pain were also reduced in the TARE group (P = 0.006 and 0.02, respectively). Moreover, there were no significant differences in the pooled analyses of complete response rates, fatigue, nausea/vomiting, 3-year OS, or 5-year OS between these groups (P = 0.24, 0.69, 0.15, 0.73, and 0.38, respectively). Significant heterogeneity was detected for endpoints including fatigue, nausea/vomiting, fever, abdominal pain, OS duration, and 3-year OS (*I^2^* = 89%, 82%, 72%, 90%, 96%, and 66%, respectively). All endpoints exhibited no significant risk of publication bias.

**Conclusions:**

This study revealed that relative to TACE, TARE performed using ^90^Y can yield significantly higher treatment response rates and prolong HCC patient survival with fewer treatment-related side effects.

The PRISMA guidelines were used to guide the execution and publication of this meta-analysis. The study is registered at INPLASY.COM (No. INPLASY202380017).

**Systematic review registration:**

INPLASY.COM, identifier INPLASY202380017.

## Introduction

Hepatocellular carcinoma (HCC) is among the most common cancers with a high mortality rate globally (1, 2). Although curative surgical tumor resection is associated with a good HCC patient prognosis, most patients are diagnosed when the disease is relatively advanced, when liver reserve capacity is limited, or when liver transplantation is unavailable; therefore, only < 30% of patients undergo surgical treatment (3–5). For patients who cannot undergo curative surgery, various locoregional or systemic treatment strategies are instead used to prolong survival and improve quality of life (1, 2, 6).

The locoregional or systemic treatment strategies are usually chosen by the Barcelona Clinic Liver Cancer (BCLC) stages (3, 5). For BCLC stage C HCC, systematic therapy is regarded as the initial course of treatment (1, 2). Recently, combined systemic treatment based on tyrosine kinase inhibitors plus immune checkpoint inhibitors has become the most commonly used systematic treatment for HCC (3).

Locoregional approaches to inoperable HCC management include transarterial chemoembolization (TACE), percutaneous ablation (PA), and the insertion of ^125^I seeds under computed tomography (CT) guidance (5, 7), with TACE being the most commonly implemented strategy. Furthermore, TACE can be used as a standard therapeutic intervention in BCLC stage A or B HCC patients (8), and has also been employed as a baseline treatment for HCC patients undergoing additional PA and ^125^I seed insertion procedures (5, 7).

Recently, the transarterial radioembolization (TARE) technique has emerged as an alternative to TACE and utilizes ^90^Y integrated into resin or glass matrix microspheres. Relative to TACE, TARE has many benefits including better patient quality of life, longer time-to-progression, higher antitumor activity of portal vein invasion cases, and the potential for neoadjuvant application before tumor resection (6, 9). However, the efficacy of TARE in treating HCC patients remains elusive.

Therefore, this meta-analysis aimed to compare the efficacy and safety of TARE and TACE in the management of inoperable HCC.

## Materials and methods

### Study selection

The PRISMA guidelines were used to guide the execution and publication of this meta-analysis. The study is registered at INPLASY.COM (No. INPLASY202380017).

To identify relevant studies, the Web of Science, PubMed, and Wanfang databases were searched for articles published from July 2023 with the following strategy: (((transarterial chemoembolization) OR (TACE)) AND ((transarterial radioembolization) OR (TARE))) AND ((hepatocellular carcinoma) OR (HCC)).

Studies eligible for inclusion:

- Study types: comparative analyses;
- Diseases: inoperable HCC patients;
- Intervention types: TARE vs. TACE;
- Languages: not limited.

Excluded studies included:

- single-arm studies;
- studies of patients undergoing TACE/TARE as a bridging procedure before surgery;
- studies comparing drug-eluting bead (DEB)-TACE and TARE procedures;
- reviews, letters, and case reports.

### Data extraction

Two investigators (3-years and 5-years’ experience in conducting meta-analysis) independently extracted relevant data from these studies, and any inconsistencies were resolved by discussion with a third investigator (8-years’ experience in conducting meta-analysis). The inconsistencies mainly occurred in the treatment-related data. Study baseline data (**Table 1**), patient baseline data (**Table 2**), and treatment-related data (**Table 3**), as were results pertaining to treatment response rates, complications, and patient overall survival (OS), were extracted from all the studies.

**Table 1:** Baseline data of the included studies.

	First author	Year	Country/Area	Design	NOS
1	Carr (11)	2010	USA	Retrospective	7
2	El Fouly (12)	2014	Germany, Egypt	Prospective non randomized controlled trial	7
3	Kim (13)	2021	South Korea	Retrospective	8
4	Kooby (14)	2010	USA	Retrospective	8
5	Moreno-Luna (15)	2013	USA	Retrospective	7
6	She (16)	2014	China (Hong Kong)	Retrospective	7
7	Soydal (17)	2016	Turkey	Retrospective	8
8	Yu (18)	2022	China (Hong Kong)	Retrospective	7

NOS, Newcastle-Ottawa Scale.

**Table 2:** Baseline data of the patients in the included studies.

Author	Groups	Patients (n)	Age (y)	Gender (M/F)	Etiology	MELD score	BCLC stages	Number of tumors (solitary/multiple)
Carr (11)	TARE	99	Not given	70/29	HBV, HCV, Alcohol	Not given	Not given	Not given
	TACE	691	Not given	518/173	HBV, HCV, Alcohol	Not given	Not given	Not given
El Fouly (12)	TARE	44	66.1	36/8	HBV, HCV, Alcohol, Others	9	B	0/44
	TACE	42	58.3	38/4	HBV, HCV, Alcohol, Others	10	B	10/32
Kim (13)	TARE	54	58	45/9	HBV, HCV, Alcohol, Others	Not given	A-C	Not given
	TACE	84	60	70/14	HBV, HCV, Alcohol, Others	Not given	A-C	Not given
Kooby (14)	TARE	27	58.7	23/4	HCV, Others	10.0	Not given	12/15
	TACE	44	61.0	36/8	HCV, Others	10.4	Not given	25/19
Moreno-Luna (15)	TARE	61	64	49/12	HCV, Alcohol, Others	9	A-C	13/48
	TACE	55	66	43/11	HCV, Alcohol, Others	9	A-C	20/35
She (16)	TARE	16	55	15/1	HBV	7.5	Not given	7/9
	TACE	16	62.5	13/3	HBV	8.5	Not given	6/10
Soydal (17)	TARE	40	62.3	33/7	Not given	Not given	B, C	19/21
	TACE	40	66.2	34/6	Not given	Not given	B, C	17/23
Yu (18)	TARE	17	57	13/4	HBV, HCV, Others	Not given	Not given	12/5
	TACE	54	59.5	51/3	HBV, HCV, Others	Not given	Not given	30/24

M, male; F, female; BCLC, Barcelona Clinic Liver Cancer; MELD, model for end-stage liver disease; TACE, transarterial chemoembolization; TARE, transarterial radioembolization.

**Table 3:** Data of the treatments.

Author	Groups	Embolization materials	Treatment sessions	Follow-up (months)
Carr (11)	TARE	Yttrium 90 microsphere	1.3	Not given
	TACE	Cisplatin	2.5	
El Fouly (12)	TARE	Yttrium 90 microsphere	1.4	Not given
	TACE	Doxorubicin	2.2	
Kim (13)	TARE	Yttrium 90 microsphere	Not given	27.6
	TACE	Doxorubicin	Not given	
Kooby (14)	TARE	Yttrium 90 microsphere	Not given	6
	TACE	Doxorubicin, mitomycin	Not given	
Moreno-Luna (15)	TARE	Yttrium 90 microsphere	Not given	Not given
	TACE	Doxorubicin, mitomycin	Not given	
She (16)	TARE	Yttrium 90 microsphere	Not given	Not given
	TACE	Cisplatin	Not given	
Soydal (17)	TARE	Yttrium 90 microsphere	1	53
	TACE	Mitomycin	2.8	
Yu (18)	TARE	Yttrium 90 microsphere	Not given	Not given
	TACE	Cisplatin	Not given	

TACE, transarterial chemoembolization; TARE, transarterial radioembolization.

### Evaluation of study quality

For randomized controlled trials (RCTs), study quality was evaluated using the Cochrane risk-of-bias tool. Furthermore, each item (performance, attrition, detection, selection, reporting, and other biases) was judged to exhibit a high, low, or unclear risk of bias.

For non-RCTs, the Newcastle-Ottawa scale (NOS) was employed to score studies based on the criteria of selection (4 points), exposure (3 points), and comparability (2 points). A score of ≥ 7 indicated a high-quality study.

### Endpoints

For pooled analyses, the primary endpoint was the total response rate. Whereas secondary endpoints included disease control rates, complete response rates, complications, OS duration, and 1-, 3-, and 5-year OS rates. The mRECIST criteria were used to evaluate treatment response rates (10). The detailed classification of complete response (CR), partial response (PR), stable disease (SD), and progressive disease (PD) are provided in **Supplementary Table S1**. The total response rate was computed by summing CR and PR rates, while the disease control rate was determined by summing the CR, PR, and SD rates.

### Statistical analyses

RevMan v5.3 and Stata v12.0 were used to perform these analyses. Pooled analyses of OS were performed by calculating hazard ratio (HR) values, while pooled odds ratios (ORs) and 95% confidence intervals (CIs) were determined when comparing categorical variables. Heterogeneity was evaluated via the Q test and the *I^2^* statistic. Random-effects models were used in cases of significant heterogeneity (*I^2^* > 50%), otherwise, fixed-effects models were employed. The causes of heterogeneity were assessed *via* sensitivity analyses in which articles were individually excluded from pooled analyses. Funnel plots were generated to gauge the potential for publication bias, and this risk was considered low when all studies fell within the established plots. Egger’s test was performed to assess publication bias in cases where funnel plots could not exclude potential biases. P < 0.05 was set as the cut-off to define significance.

## Results

### Study selection

**Figure 1:**
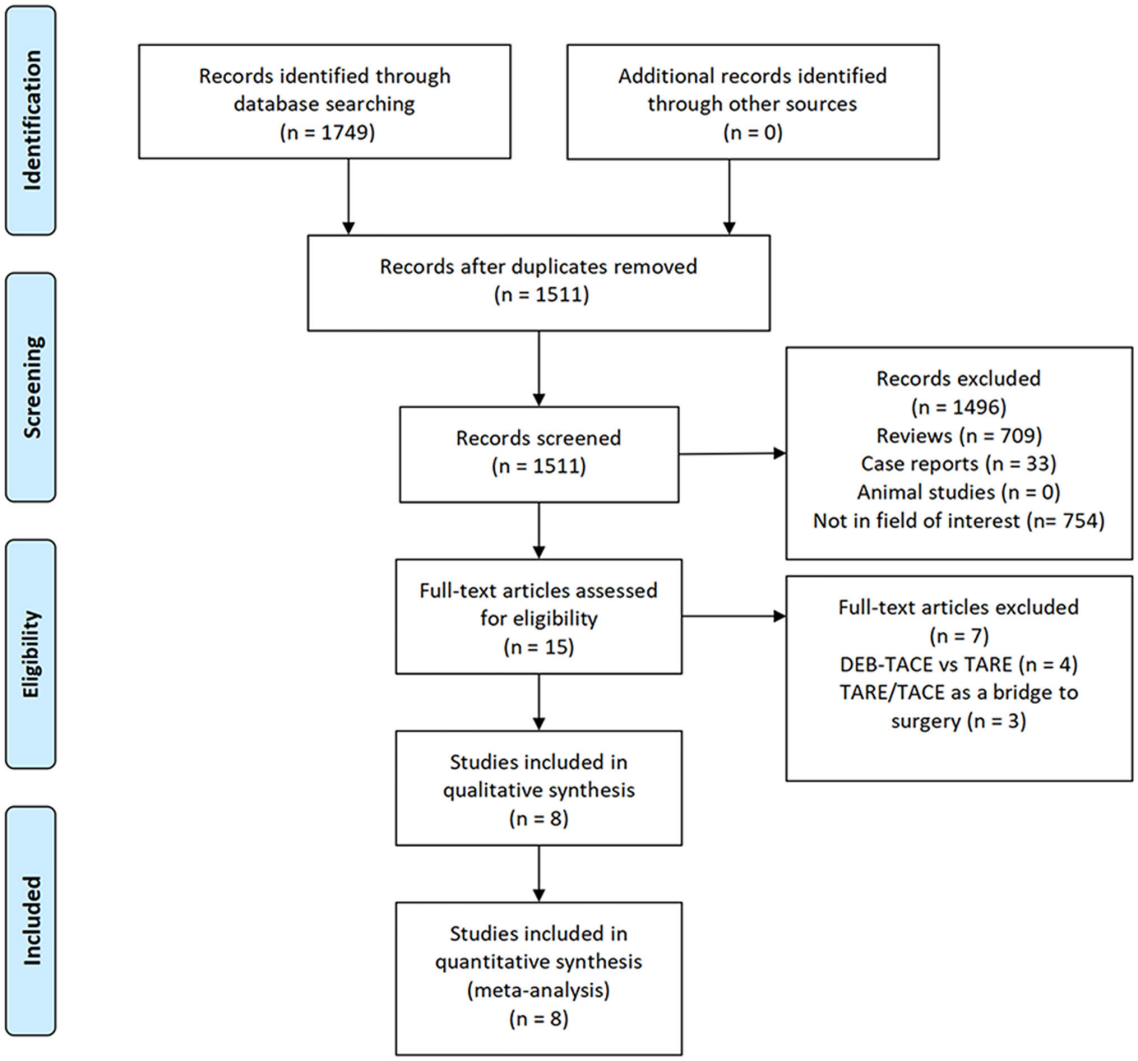
The study selection process for this meta-analysis.

An initial literature search identified 1,749 potentially relevant studies, however, based on the defined criteria, 8 studies (11–18) were enrolled in the final meta-analysis (**Figure 1**). These 8 publications included 7 retrospective studies (11, 13–18) and 1 prospective non-RCT (12). These articles were published between 2010 and 2022 by research teams in Asia, Europe, North America, and Africa. All articles were of high quality with NOS scores from 7-8 (**Supplementary Table S2**). The patient populations in these articles included 358 and 1,026 HCC patients who respectively underwent TARE and TACE procedures. ^90^Y microspheres were used to perform all TARE procedures, while TACE procedures were performed using combinations of lipiodol with mitomycin, cisplatin, or doxorubicin.

### CR rates

**Figure 2:**
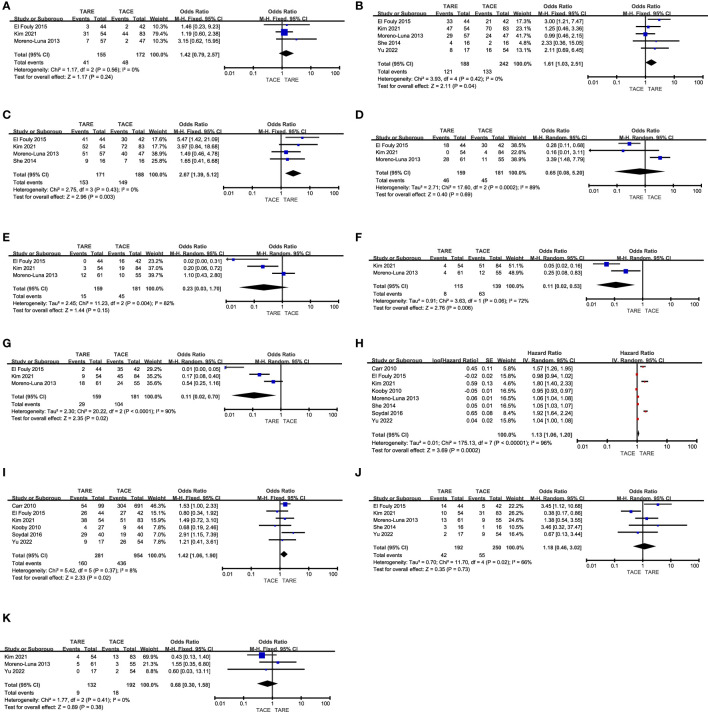
The comparative results of **(A)** CR rates, **(B)** total response rates, **(C)** disease control rates, **(D)** fatigue rates, **(E)** nausea/vomiting rates, **(F)** fever rates, **(G)** abdominal pain rates, **(H)** OS, **(I)** 1-year OS rates, **(J)** 3-year OS rates, **(K)** 5-year OS rates.

CR rates were reported in 3 studies (12, 13, 15) comprising 327 patients (TARE: 155, TACE: 172). Pooled CR rates were similar in both of these groups (26.5% vs. 27.9%, P = 0.24, **Figure 2A**), and no heterogeneity was detected (*I^2^* = 0%).

### Total response rates

In total 5 studies (12, 13, 15, 16, 18) with 430 patients (TARE: 188, TACE: 242) reported total response rates. The TARE group patients indicated a significantly higher pooled total response rate (64.4% vs. 55.0%, P = 0.04, **Figure 2B**), and no heterogeneity was detected (*I^2^* = 0%).

### Disease control rates

Disease control rates were reported in 4 studies (12, 13, 15, 16) analyzing 359 patients (TARE: 171, TACE: 188). A significantly higher pooled disease control rate was observed in the TARE group as compared to the TACE group (89.5% vs. 79.3%, P = 0.003, **Figure 2C**), and no heterogeneity was detected (*I^2^* = 0%).

#### Fatigue

Rates of patient fatigue were reported in 3 studies (12, 13, 15), containing 159 TARE and 181 TACE group patients, respectively. These two groups indicated comparable pooled fatigue rates (28.9% vs. 24.9%, P = 0.69, **Figure 2D**). Significant heterogeneity was observed (*I^2^* = 89%), however, omitting Moreno-Luna et al. study eliminated this heterogeneity (*I^2^* = 0%) (15). When this study was not included in the pooled analysis, TARE patients indicated a significantly lower pooled fatigue rate than TACE patients (P = 0.002).

#### Nausea and vomiting

Nausea and vomiting rates were reported in 3 studies (12, 13, 15), analyzing 159 TARE and 181 TACE groups patients. Furthermore, both the groups indicated similar pooled nausea and vomiting rates (9.4% vs. 24.9%, P = 0.15, **Figure 2E**). While significant heterogeneity was detected (*I^2^* = 82%), sensitivity analyses could not identify its source.

#### Fever

Fever rates were reported in 2 studies (13, 15), enrolling 115 and 139 patients who underwent TARE and TACE, respectively. A significantly lower pooled fever rate was detected for patients who underwent TARE relative to TACE (7.0% vs. 45.3%, P = 0.006, **Figure 2F**). Moreover, significant heterogeneity was detected (*I^2^* = 72%). There were insufficient studies to perform a sensitivity analysis.

#### Abdominal pain

The incidence of abdominal pain was reported in 3 studies (12, 13, 15), comprising 159 and 181 patients in the TARE and TACE groups, respectively. Patients who underwent TARE had a significantly lower pooled abdominal pain rate relative to TACE (18.2% vs. 57.5%, P = 0.02, **Figure 2G**). Moreover, there was significant heterogeneity was detected (*I^2^* = 90%) and sensitivity analyses could not identify its source.

### OS

The OS duration for enrolled patients was reported in all studies, and the forest plots revealed a significantly longer pooled OS for patients who underwent TARE than those who underwent TACE (HR: 1.13, 95% CI: 1.06-1.20, P = 0.0002, **Figure 2H**). There was significant heterogeneity between the groups (*I^2^* = 96%), sensitivity analyses could not identify its source.

#### 1-year OS

Patient 1-year OS rates were reported in 6 studies (11–14, 17, 18), containing 281 and 951 patients in the TARE and TACE groups, respectively. TARE patients indicated a significantly higher pooled 1-year OS rate than TACE patients (56.9% vs. 45.7%, P = 0.02, **Figure 2I**), and no heterogeneity was detected (*I^2^* = 8%).

#### 3-year OS rate

Patient 3-year OS rates were reported in 5 studies (12, 13, 15, 16, 18), comprising 192 and 250 patients in the TARE and TACE groups, respectively. The pooled 3-year OS rates were comparable in both groups (21.9% vs. 22.0%, P = 0.73, **Figure 2J**), and there was significant heterogeneity (*I^2^* = 66%), which was reduced (*I^2^* = 8%) by omitting the Kim et al. study (13). Pooled analyses without this study revealed that the 3-year OS rates for patients in the TACE and TARE group patients were similar (P = 0.10).

#### 5-year OS rates

Patient 5-year OS rates were reported in 3 studies (13, 15, 18), analyzing 132 and 192 patients in the TARE and TACE groups, respectively. The pooled 5-year OS rates were comparable in both groups (6.8% vs. 9.4%, P = 0.38, **Figure 2K**), and no heterogeneity was detected (*I^2^* = 0%).

### Publication bias

Funnel plots revealed no evidence of significant publication bias for the CR rate, total response rate, disease control rate, fever, 1-year OS, or 5-year OS endpoints (**Supplementary Figures S1A-K**). The Egger’s test evaluated the remaining endpoints and revealed no significant publication bias for the fatigue, nausea/vomiting, abdominal point, OS, or 3-year OS endpoints (P = 0.76, 0.252, 0.213, 0.099, and 0.45).

## Discussion

This meta-analysis was designed to evaluate the safety and clinical efficacy of TARE and TACE as treatment strategies to manage inoperable HCC based on treatment responses, complications, and survival outcomes for affected patients.

Treatment response rates are a key determinant of HCC patient prognosis, serving as an important short-term outcome for evaluating a therapeutic strategy. Pooled CR rates in the present meta-analysis were similar in the TARE and TACE patient groups, although the total response and disease control rates in the TARE group were higher than those in the TACE group. This suggests that TARE procedures performed using ^90^Y can more readily take advantage of the vascular nature of HCC tumors to induce necrotic cell death upon ablation (19). These benefits may be attributed to the high dose of absorbed radiation, the state of the liver, the goals of each therapeutic approach, and the target volume for treated patients. The delivery of high radiation doses to the tumor capillary bed can more significantly induce necrosis following TARE than after TACE treatment (20, 21).

Compared to external radiation therapy, brachytherapy has various advantages due to the direct contact between the radiation source and internal tumor regions, enabling the persistent delivery of radiotherapy while minimizing off-target damage (21). Here, CR, total response, and disease control rates indicated low heterogeneity (*I^2^* = 0%), suggesting the credibility of these findings. Furthermore, the 89.5% pooled disease control role for the TARE group was also similar to the 91.1% rate reported previously in a meta-analysis comparing TACE and CT-guided ^125^I seed insertion in HCC patients (22). Although CT-guided ^125^I seed insertion has been employed as a brachytherapy-based approach to HCC patient treatment (7, 22), this technique carries a risk of hematoma and pneumothorax (22). TARE, however, is performed within tumor-feeding arteries and thus avoids the potential for such complications (16). TARE procedures can be implemented more easily than TACE or CT-guided ^125^I seed insertion.

The present analyses revealed that TARE treatment was associated with lower pooled rates of fever and abdominal pain than TACE. TARE entails the injection of radioactive particles into a target artery in the liver without occluding that artery (23). Furthermore, this approach does not induce VEGF or HIF-1a overexpression, which can cause fever and pain in patients (12). This study also observed no differences in nausea/vomiting or fatigue between these groups, suggesting that TARE cannot abrogate gastrointestinal or hepatic toxicity associated with this locoregional treatment.

There were substantial variations in outcomes comparing OS durations were observed among studies, however, the pooled data suggested that TARE is associated with the significant prolongation of OS relative to TACE. The 1-year survival rate in the TARE group was also superior to that in the TACE group, which might be associated with the higher treatment response rate in this group. Whereas no significant differences were observed in pooled 3-year (21.9% vs. 22.0%) or 5-year (6.8% vs. 9.4%) OS rates between these groups. The low 3 and 5-year OS rates in both groups highlight the limitations of TARE and TACE to control HCC over extended periods.

There are some limitations to the present study. For one, this meta-analysis did not include any RCTs and the results are subject to a high risk of bias. In the future, appropriately designed prospective RCTs should be performed to validate the present results. Moreover, these studies did not employ uniform TACE protocols for the medication types or dosages, potentially contributing to further bias. Third, the numbers of patients in the TARE and TACE groups were not balanced, and there were variations in the etiological basis for HCC across the included studies. All of these factors may have further contributed to bias with respect to these results.

## Conclusion

In summary, this meta-analysis showed that relative to TACE, TARE performed using ^90^Y can achieve better treatment response rates and OS benefits in inoperable HCC patients while causing fewer side effects. However, this meta-analysis was conducted mainly based on the retrospective study. Therefore, the treatment effectiveness (such as treatment response, survival function, and safety) of TARE for inoperable HCC should be corroborated by future randomized controlled trials.

## Data Availability

The original contributions presented in the study are included in the article/**Supplementary Material**. Further inquiries can be directed to the corresponding author/s.

## Supplementary material

Supplementary Figure 1 The funnel plots of **(A)** CR rates, **(B)** total response rates, **(C)** disease control rates, **(D)** fatigue rates, **(E)** nausea/vomiting rates, **(F)** fever rates, **(G)** abdominal pain rates, **(H)** OS, **(I)** 1-year OS rates, **(J)** 3-year OS rates, **(K)** 5-year OS rates.
