# Supplementary material for: Transarterial radioembolization versus chemoembolization for hepatocellular carcinoma: a meta-analysis
Source: Front Oncol. 2025 Jan 17;14:1511210. doi: 10.3389/fonc.2024.1511210 (PMC11782047; doi:10.3389/fonc.2024.1511210)
Supplement: Supplementary file 12 [file Table1.doc]

Table 1 Modified Response Evaluation Criteria in Solid Tumors for HCC.

|  | Definitions |
| --- | --- |
| Complete response | Disappearance of any intratumoral arterial enhancement in all target lesions |
| Partial response | At least a 30% decrease in the sum of diameters of viable (enhancement in the arterial phase) target lesions, taking as reference the baseline sum of the diameters of target lesions |
| Stable disease | Any cases that do not qualify for either partial response or progressive disease |
| Progression disease | An increase of at least 20% in the sum of the diameters of viable (enhancing) target lesions, taking as reference the smallest sum of the diameters of viable (enhancing) target lesions recorded since treatment started |

HCC: hepatocellular carcinoma.
