# Supplementary material for: Transarterial radioembolization versus chemoembolization for hepatocellular carcinoma: a meta-analysis
Source: Front Oncol. 2025 Jan 17;14:1511210. doi: 10.3389/fonc.2024.1511210 (PMC11782047; doi:10.3389/fonc.2024.1511210)
Supplement: Supplementary file 13 [file Table2.doc]

Supplement Table 2. Assessment of non-randomized study quality.

| Studies | Quality Indicators From Newcastle-Ottawa Scale | | | | | | | | Total score |
| --- | --- | --- | --- | --- | --- | --- | --- | --- | --- |
|  | Selection | | | | Comparability | Outcome | | |  |
|  | Representativeness of the exposed cohort | Selection of the non exposed cohort | Ascertainment of exposure | Demonstration the outcome of interest was not present at start of study | Comparability of cohorts on the basis of the design or analysis | Assessment of outcome | Was follow-up long enough for outcomes to occur | Adequacy of follow up of cohorts |  |
| Carr [11] | * |  | * | * | * | * | * | * | 7 |
| El Fouly [12] | * |  | * | * | * | * | * | * | 7 |
| Kim [13] | * | * | * | * | * | * | * | * | 8 |
| Kooby [14] | * | * | * | * | * | * | * | * | 8 |
| Moreno-Luna [15] | * |  | * | * | * | * | * | * | 7 |
| She [16] | * |  | * | * | * | * | * | * | 7 |
| Soydal [17] | * | * | * | * | * | * | * | * | 8 |
| Yu [18] | * |  | * | * | * | * | * | * | 7 |
